# Point-of-Care Placental Growth Factor for Predicting Adverse Outcomes in Sierra Leone

**DOI:** 10.1161/HYPERTENSIONAHA.125.24526

**Published:** 2025-08-05

**Authors:** Katy Kuhrt, Rossetta Cole, Moses M’Bayoh, Chileshe Mabula-Bwalya, Alice Hurrell, Alexandra Ridout, Cristina Fernandez-Turienzo, Paul T. Seed, Lucy C. Chappell, Kate Bramham, Andrew H. Shennan

**Affiliations:** Department of Women and Children’s Health, King’s College London, St Thomas’s Hospital, London, United Kingdom (K.K., A.H., A.R., C.F.-T., P.T.S., L.C.C., K.B., A.H.S.).; Department of Reproductive and Child Health, Princess Christian Maternity Hospital, Ministry of Health and Sanitation, Freetown, Sierra Leone (R.C., M.M.).

**Keywords:** eclampsia, maternal mortality, point-of-care systems, pre-eclampsia, pregnancy

## Abstract

**BACKGROUND:**

PlGF (placental growth factor) concentrations are lower in preeclampsia, a major cause of maternal death; testing improves diagnosis and outcomes. We evaluated 2 novel, whole blood, point-of-care PlGF tests (RONIA and Lepzi Quanti) in a low-resource setting for predicting adverse outcomes.

**METHODS:**

A prospective observational cohort study was conducted in women with hypertension in Sierra Leone, each of which were 24 to 36^+6^ weeks of gestation. Eligible women underwent concealed RONIA and Lepzi Quanti PLGF testing. Optimal rule-out and rule-in thresholds were determined for predicting maternal (death and eclampsia) and perinatal (stillbirth, termination pre-viability, and neonatal death) composite outcomes. Sensitivity, specificity, negative predictive values, and positive predictive values were assessed.

**RESULTS:**

Analysis included women with RONIA (n=488) and Lepzi Quanti (n=140) PlGF tests. Optimal thresholds were >60 or >90 pg/mL (rule-out) and <20 or <12 pg/mL (rule-in) for RONIA and Lepzi Quanti, respectively. For tests <34 weeks, RONIA <60 pg/mL had high sensitivity and negative predictive value for ruling out maternal (94.9% and 94.6%) and perinatal (100%) composite outcomes. RONIA <20 pg/mL indicated higher risk (positive predictive values, 16.3% and 40.9% respectively). Lepzi Quanti <90 pg/mL had 100% sensitivity and negative predictive value for ruling out maternal and perinatal composites. Conversely, with Lepzi Quanti <12 pg/mL, nearly a quarter and a half of pregnancies experienced the maternal and perinatal composites (positive predictive values, 22.0% and 48.0%). Performance declined slightly at later gestations.

**CONCLUSIONS:**

Point-of-care PlGF testing shows accurate rule-out performance for serious outcomes, with potential for risk stratification in low-resource settings. For both tests, a minority of cases fell into an intermediate category, which would warrant increased surveillance to determine progress.

**NOVELTY AND RELEVANCE**

**What Is New?**

For the first time, we have prospectively demonstrated that 2 novel whole blood point-of-care PlGF (placental growth factor) tests can accurately rule out maternal death, eclampsia, and stillbirth in women with suspected preeclampsia in a low-resource setting.

**What Is Relevant?**

Preeclampsia accounts for 25% of maternal deaths in low- and middle-income countries and is linked to a 2- to 6-fold increased risk of hypertension and cardiovascular disease. Most deaths are preventable with early detection and risk stratification to enable timely management.

**Clinical/Pathophysiological Implications?**

Point-of-care PlGF can accurately rule out serious preeclampsia-related adverse pregnancy outcomes and could support clinicians to plan the timing of delivery, especially valuable in settings with limited neonatal resources.

Preeclampsia and other hypertensive disorders of pregnancy affect 10% of pregnancies^1^ and are a leading cause of maternal death. It is estimated that around 40 000 maternal deaths and 500 000^1^ neonatal deaths annually are associated with preeclampsia, the majority occurring in low- and middle-income countries (LMICs). Most deaths can be avoided with early identification, enabling timely management (antihypertensives, anticonvulsants, and appropriately planned birth).

Preeclampsia is often asymptomatic, even in the presence of severe disease,^2^ making diagnosis challenging, particularly in LMICs, due to limited access to blood pressure (BP) monitoring equipment, urine dipsticks, blood tests, and ultrasound scans to assess fetal well-being. Even when diagnostics are available, BP and urine have demonstrated poor sensitivity for predicting adverse pregnancy outcomes (18%–36%) in a large US-based cohort study,^3^ limiting their clinical utility to stratify risk and determine management. While typically underresearched in LMICs, this is likely generalizable.

The advent of angiogenic biomarker testing, such as PlGF (placental growth factor) alone and sFlt-1 (soluble fms-like tyronsine kinase)/PlGF ratio, has transformed the ability to identify high-risk women,^4,5^ and PLGF-based testing is now incorporated into the National Institute for Health and Care Excellence national UK guidelines^6^ for management of women with suspected preeclampsia and into International Society for the Study of Hypertension in Pregnancy guidelines in the definition of preeclampsia.^7^

The few PlGF studies that have been undertaken in LMICs include a blinded prospective cohort study^8^ and an operational pilot study of plasma PlGF measurement^9^ in women presenting with suspected preeclampsia in Mozambique, where low PlGF concentration was significantly associated with shorter time to delivery and adverse pregnancy outcomes, and a small pilot study in India where 50 women with hypertension underwent third-trimester SfLT-1/PlGF and high risk ratios were associated with more severe preeclampsia and pregnancy complications.^10^

However, to date, PlGF-based measurements for routine clinical use and study settings, including in LMICs, are not suitable for point of care (POC) and require centrifugation. This is prohibitive to sustainable implementation in Sierra Leone and other LMICs. RONIA is a small, POC-PlGF platform, using innovative upconverting nanoparticle technology, which enables high-sensitivity, quantitative immunoassays from a single drop of whole blood (20 µL), with a result in 30 minutes. Lepzi Quanti PlGF is a small, portable, fluorescence immunoassay for quantitative determination of PlGF in ethylenediaminetetraacetic acid anticoagulated whole blood or plasma samples, requiring 200 µL of venous whole blood, with a result in 15 minutes. Our objective was to evaluate the prognostic test performance of novel whole blood RONIA and Lepzi Quanti PlGF devices for serious adverse pregnancy outcomes in a low-resource setting.

## Methods

### Data Availability

The data that support the findings of this study are available from the corresponding author upon reasonable request.

### Study Design

This prospective observational cohort study was undertaken between June 2022 and April 2024 in a tertiary government maternity hospital in Freetown, Sierra Leone. Consecutive women aged ≥16 years presenting or referred with symptoms or signs of suspected or actual preeclampsia between 24^+0^ to 36^+6^ weeks of gestation, with a singleton or twin pregnancy, were eligible. Participants were included when the attending health care provider deemed that the women required evaluation for preeclampsia, with symptoms or signs such as headache, visual disturbances, epigastric or right upper quadrant pain, hypertension, dipstick protein, or suspected fetal growth restriction. Written study information was provided and explained verbally, and participants signed or marked the consent form by thumbprint for those unable to write. BP measurement was taken as part of routine care, with women sitting, and with the right arm supported at the level of the heart, using a pregnancy-validated device. BP measurement was repeated if hypertension was detected on the first reading, and the second reading was recorded. Normotensive readings were not repeated.

All women underwent RONIA testing. During the study period, we had an additional opportunity to collect samples for Lepzi Quanti PlGF, and therefore, a subset of women had both PlGF tests. For the RONIA test, 20 µL of whole blood was collected by finger prick, and for the Lepzi Quanti PlGF test, 200 µL of whole blood was collected by venous draw according to the manufacturers’ instructions. Both samples were immediately analyzed using RONIA and Lepzi Quanti readers, respectively, to determine PlGF concentration. Results were concealed from the clinical team. Pregnancy outcome details for the woman and infant were obtained from case note review, and data quality checks were performed by an external researcher, including adjudication by an obstetrician or neonatologist. The study was approved by King’s College London Research Ethics Committee (RECSM22/23-22669) and Sierra Leone Ethics and Scientific Review Committee (10/03/2022).

The prespecified primary maternal outcome was a composite of maternal death or eclampsia. Additional maternal outcomes are given as follows: individual components of the composite; parameters defined in the miniPIERS consensus, where available and reported in this setting (excluding blood transfusion as this was commonly performed for reasons other than preeclampsia-related)^11^: early delivery (<37 weeks of gestation, within 7 and 14 days of test; severe hypertension (BP ≥160/110 mm Hg); high dependency unit admission; use of magnesium sulfate; and use of intravenous antihypertensives. Perinatal outcomes included a composite of 1 or more of stillbirth, neonatal death before discharge, or termination previability (to protect maternal life). Additional perinatal outcomes are given as follows: components of the composite; mode of delivery; gestational age at delivery (by best clinical estimate); birthweight; birthweight centile; neonatal unit admission; sepsis with clinical evidence of infection; antibiotics for possible serious bacterial infection; Apgar score at 5 and 10 minutes; clinical diagnosis of hypoxic ischemic encephalopathy (low Apgar score at birth, no crying, and need for resuscitation); neonatal seizures; respiratory distress syndrome; supplementary oxygen required: use of continuous positive airway pressure ventilation; clinical diagnosis of necrotizing enterocolitis (abdominal distension with a history of formula feeding); hypoglycemia (<2.6 mmol) requiring intervention; hypothermia (single documented temperature <36.5 °C); neonatal jaundice requiring phototherapy; and nasogastric feeding.

### Statistical Analysis

Women were classified according to their gestation at the time of the PlGF test, <34 weeks of gestation, and 34 to 36^+6^ weeks of gestation based on the best clinical estimate. Descriptive statistics were used to summarize the demographic data, and POC-PlGF results were presented using mean (SD) or median (interquartile range) depending on distribution. Optimal rule-in and rule-out cut-points were selected from a range of thresholds taking into account the balance between sensitivity and specificity and prediction of maternal and perinatal composite outcomes. To determine the prognostic performance for these and other predefined end points, sensitivity, specificity, positive predictive values (PPVs), and negative predictive values (NPVs) were calculated based on the selected rule-in and rule-out thresholds, and the area under the receiver operating characteristic curve was generated. Median and interquartile ranges from time from POC-PlGF testing to delivery were calculated. We did a sensitivity analysis on the primary maternal and perinatal outcomes excluding twin pregnancies. Analysis was performed with statistical support from King’s College London Life Course Sciences (STATA/SE, version 18). The study is reported in accordance with the Standards for the Reporting of Diagnostic Accuracy Studies guidelines (Figure S1).

## Results

**Figure 1.**
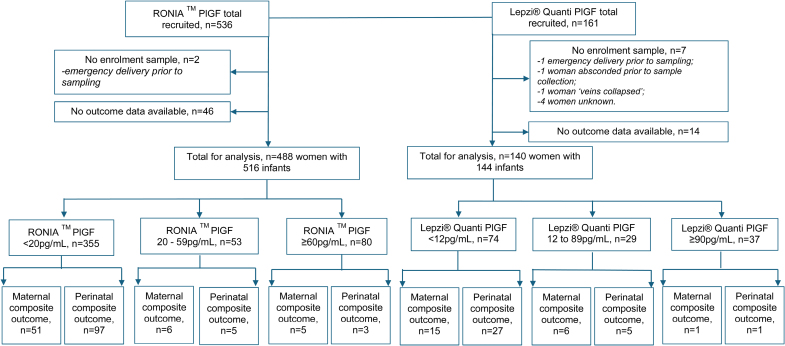
**Flow diagram of participants through the study.** PlGF, placental growth factor.

**Figure 2.**
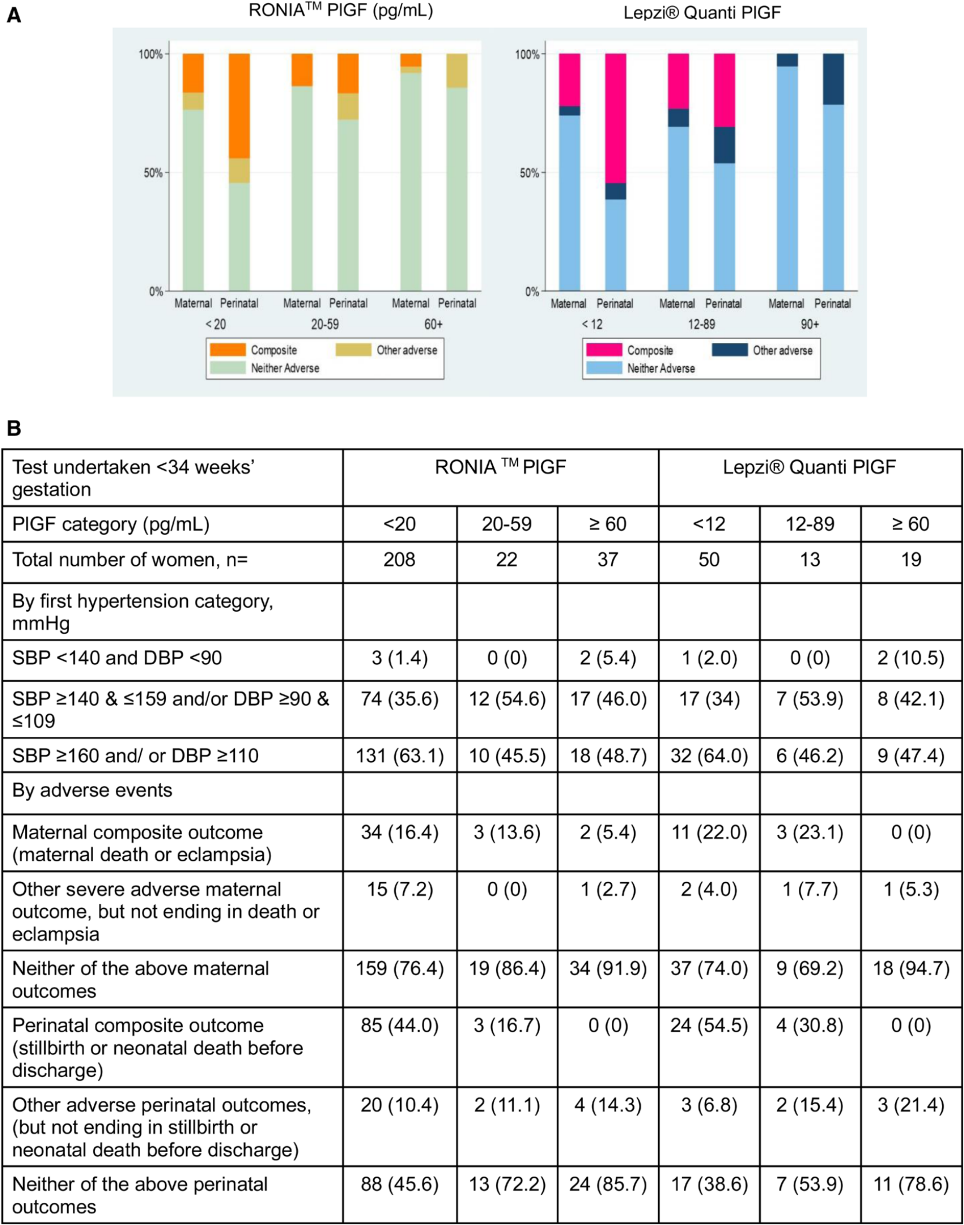
**Adverse maternal and perinatal outcome stratified by PlGF (placental growth factor) category in women with tests done at <34 weeks’ gestation for RONIA^TM^ and Lepzi^®^ Quanti PlGF. A**, Final maternal and perinatal outcomes, stratified by PlGF category. **B**, Hypertension category and adverse maternal and perinatal outcomes stratified by PlGF category. DBP indicates diastolic blood pressure; and SBP, systolic blood pressure.

Five hundred thirty-six women were recruited and underwent RONIA testing; 161 women also underwent Lepzi Quanti PlGF testing. We recruited all those who were approached, eligible, and consented (Figure 1). No adverse events were reported related to performing PlGF testing. Outcome measures were collected in full on 488 of 536 and 140 of 161 occasions for RONIA and Lepzi Quanti tests, respectively. Women without a valid PlGF result (RONIA, n=2; LEPZI Quanti, n=7) and those lost to follow-up (RONIA, n=46; LEPZI Quanti, n=14) were excluded. The characteristics of the remaining women are shown in Table 1, along with maternal and perinatal outcomes, with additional adverse maternal and perinatal outcomes in Tables S1 and S2, respectively. Proportions of women by hypertension category and maternal and perinatal adverse events stratified by PlGF are shown in Figure 2. The diagnostic accuracy of PlGF for predicting maternal and perinatal composite outcomes is shown in Tables 2 and 3 based on our selected thresholds: <20 and <60 pg/mL for RONIA PlGF and <12 and <90 pg/mL for Lepzi Quanti PlGF.

**Table 1.**
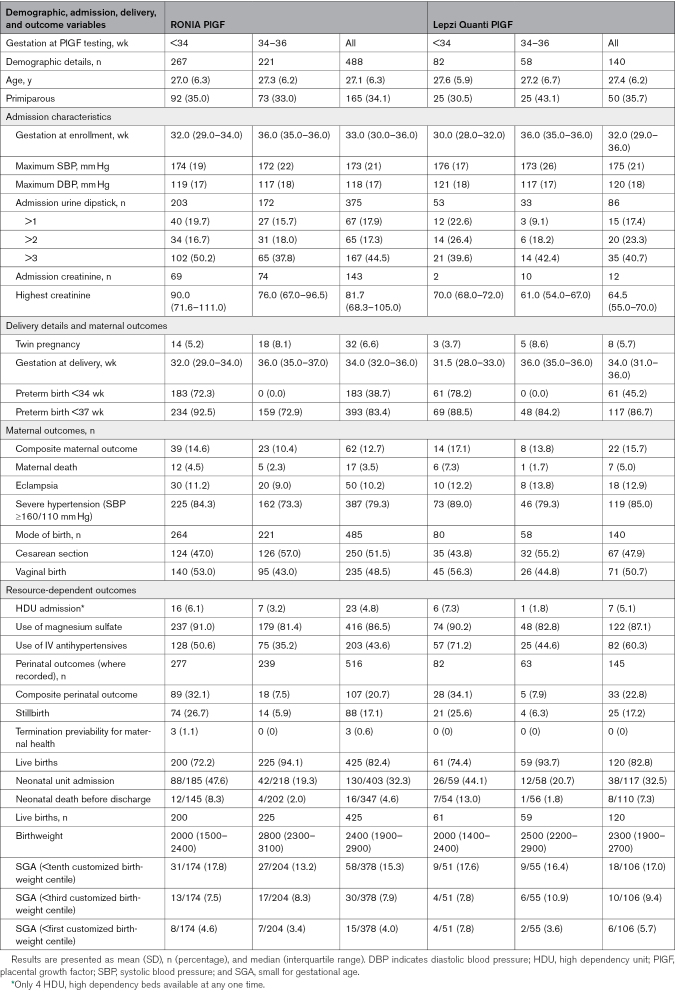
Maternal Demographics, Admission Characteristics, Delivery Details, and Maternal and Perinatal Outcomes

**Table 2.**
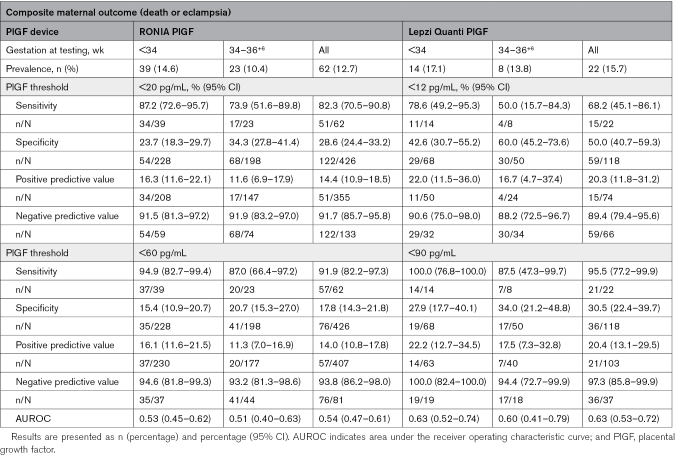
RONIA and Lepzi Quanti PlGF Prediction of Composite Maternal Outcome Based on PlGF Thresholds of <20 and <60 pg/mL, and <12 and <90 pg/mL, Respectively

**Table 3.**
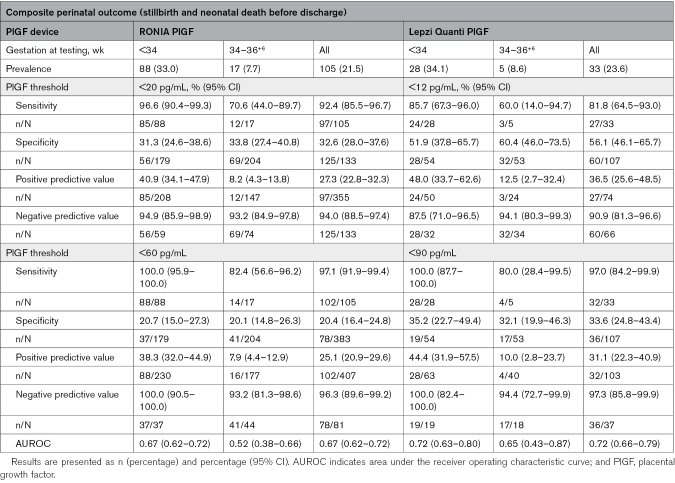
RONIA and Lepzi Quanti PlGF Prediction of Composite Perinatal Outcome Based on PlGF Thresholds of <20 and <60 pg/mL, and <12 and <90 pg/mL, Respectively

For the maternal composite (n=62), abnormal RONIA PlGF <60 pg/mL had a high sensitivity of 91.9% (95% CI, 82.2%–97.3%) and an NPV of 93.8% (95% CI, 86.2%–98.0%) for women tested at all gestations. Sensitivity and NPV for the maternal composite improved still further for women tested at <34 weeks of gestation to 94.9% (95% CI, 82.7%–99.4%) and 94.6% (95% CI, 81.8%–99.3%), respectively, and for maternal death (n=17), where sensitivity and NPV were 100%. Abnormal Lepzi Quanti PlGF <90 pg/mL had 100% sensitivity and NPV for the maternal composite (n=22; Table 2) and for individual components of the composite, declining slightly at later gestations, except for maternal death (n=7) where 100% sensitivity and NPV were maintained across all gestations (Tables S3 and S4).

For the perinatal composite outcome (n=105), abnormal RONIA PlGF <60 pg/mL had a high sensitivity of 97.1% (95% CI, 91.8%–99.4%) and an NPV of 96.3% (95% CI, 89.6%–99.2%) at all gestations tested, and these were improved in women tested <34 weeks of gestation with sensitivity and NPV of 100% (95% CI, 95.8%–100.0% and 90.5%–100%, respectively; Table 3). Lepzi Quanti PlGF had 100% sensitivity and NPV for the perinatal composite outcome (Table 3), and for the individual components of the composite for women tested at <34 weeks of gestation, declining slightly at later gestations tested, except for neonatal death before discharge, where 100% sensitivity and NPV were maintained across all gestations tested (Tables S5 and S6).

Among women who underwent RONIA testing at all gestations, there were 8 false negative cases (1 maternal death, 4 eclampsia, 1 stillbirth, and 2 neonatal deaths before discharge). The maternal death was attributed to a known seizure disorder, with a PlGF of 254.92 pg/mL, while sepsis was reported in 1 of the babies who had a neonatal death (with normal birthweight; PlGF, 692.34 pg/mL). None of the false negative eclampsia cases had borderline PlGF results: 115, 677, 102, and 697 pg/mL. For Lepzi Quanti PlGF, there were 2 false negative cases (1 eclampsia case; PlGF, 276.44 pg/mL; 1 severe adverse neonatal event; PlGF, 92.68 pg/mL). Both of these cases were verified against the source notes, but, due to limited documentation, it was not possible to determine whether additional diagnoses may have contributed to or caused their outcomes.

Test performance for additional clinical outcomes is presented in Tables S7 through S11. For both abnormal RONIA <60 pg/mL and Lepzi Quanti <90 pg/mL, sensitivity and NPV were 100% for women tested <34 weeks for SGA <third centile, albeit in small numbers of women (Table S8).

**Figure 3.**
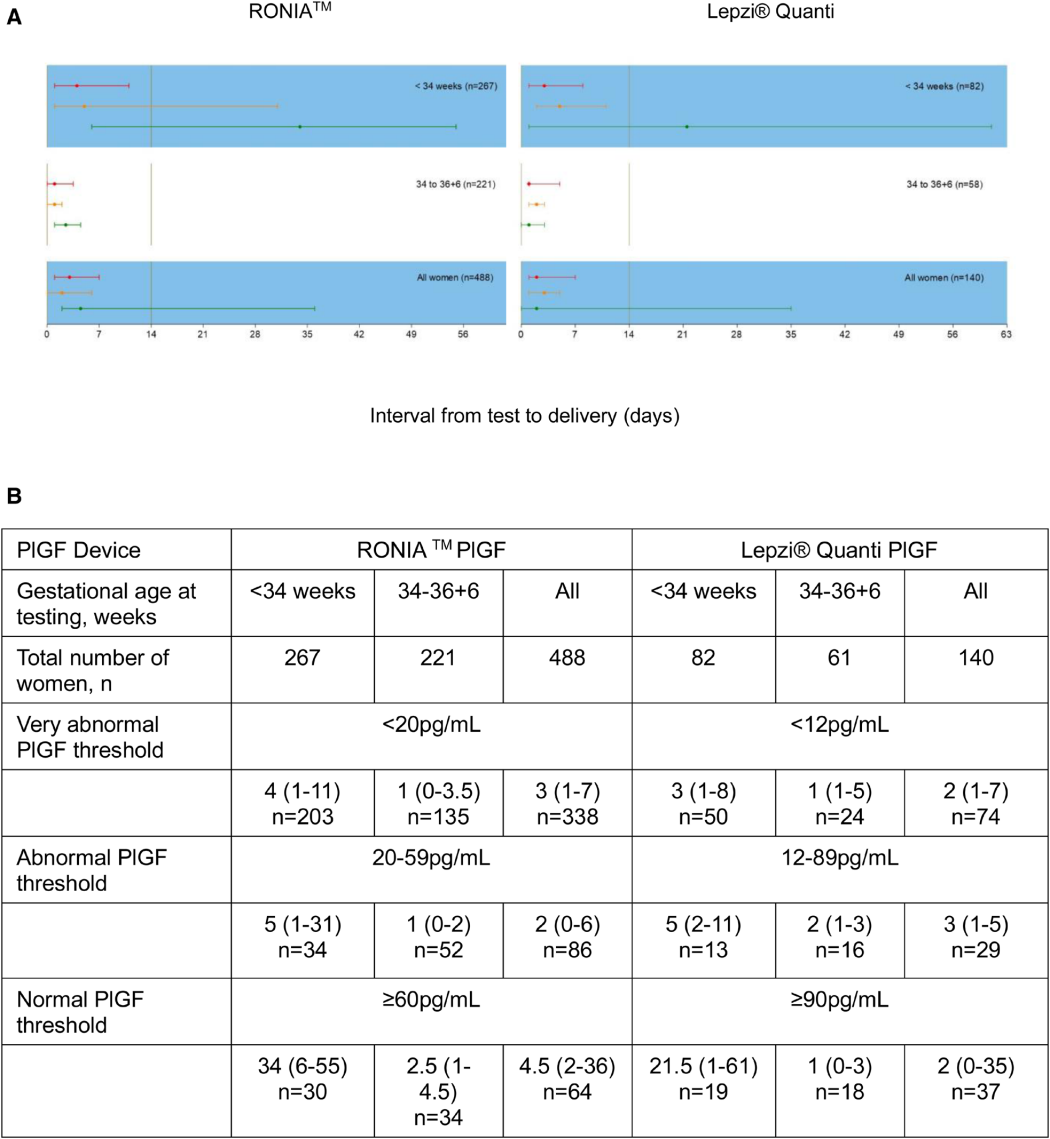
**Time to delivery (median, interquartile range) stratified by RONIA and Lepzi Quanti PlGF (placental growth factor) concentration for all participants. A**, Time to delivery (median, IQR) stratified by RONIA^TM^ and Lepzi^®^ Quanti PlGF concentration for all participants. **B**, Time to delivery (median, IQR) stratified by RONIA^TM^ and Lepzi^®^ Quanti PlGF concentration for all gestational age categories at testing.

For RONIA PlGF <20 pg/mL, PPV for delivery within 14 days was 88.0% (95% CI, 84.0%–91.2%), and for preterm delivery <37 weeks of gestation, it was 87.1% (95% CI, 83.0%–90.4%), which improved to 97.4% (95% CI, 94.0%–99.1%) for women tested <34 weeks of gestation. For RONIA PlGF <60, although sensitivity was high (>90%) for both delivery within 14 days and preterm delivery <37 weeks of gestation, for women tested <34 weeks of gestation, NPV was modest due to high prevalence of the outcome (Tables S10 and S11). For Lepzi Quanti PlGF <12 pg/mL, PPV for prediction of delivery within 14 days was 87.2% (95% CI, 74.3%–95.2%) for women tested <34 weeks of gestation, improving at later gestations. For preterm delivery <37 weeks of gestation, PPV was 97.8% (95% CI, 88.5%–99.9%) for women tested <34 weeks of gestation, declining at later gestations. However, despite modest overall performance in determining time to delivery, the median time between test and delivery was markedly longer in women with a normal PlGF result for both tests (Figure 3): RONIA (34 versus 4 days) and Lepzi Quanti (21.5 versus 3 days). A prespecified sensitivity analysis excluding women with twin pregnancies showed very similar results (Tables S12 and S13).

## Discussion

Whole blood RONIA and Lepzi Quanti PlGF accurately identified women admitted with hypertension, clinical features of preeclampsia, or both who did not go on to have a serious adverse event, especially in those tested <34 weeks of gestation, in a challenging LMIC environment. For maternal and perinatal composite outcomes, this was represented by sensitivities of 94.9% and 100.0%, respectively (RONIA) and 100.0% for both (Lepzi Quanti). The median time to delivery was markedly shorter for women tested <34 weeks with abnormal RONIA and Lepzi Quanti PlGF values (compared with those with normal results).

There were 17 (3.5%) maternal deaths, 50 eclampsia cases, and 106 perinatal deaths compared with previous studies informing UK national guidelines,^4,5,12^ where no maternal mortality and few stillbirths have occurred. In the context of managing these women, we specifically wanted to rule out very severe outcomes to avoid delivery as previously stated. This is a unique analysis and different from previous studies, regarding the prediction of severe features, which would be meaningless in this setting given the relatively high prevalence of serious outcomes.

In this high-risk population, the majority of women had an abnormal PlGF. Most women with a very abnormal PlGF did not die or have eclampsia, explaining the low PPV and specificities for both tests. However, given the severity of outcomes, we accept a high false positive rate, and action, for example, early delivery, could reasonably be considered to avoid a 10% risk of maternal death and a 30% risk of stillbirth. This is in keeping with interventions across maternity care, but this test improves stratification enabling the action to be better targeted. The PPVs for the composite maternal and perinatal outcomes are similar to those presented by Duhig et al,^5^ for a less serious outcome in a high-income setting (preeclampsia requiring delivery within 14 days), which has changed clinical practice, and are likely to be informative. Furthermore, including additional severe features (eg, from mini-piers consensus) may have improved these values. However, the absence of laboratory diagnostics in the study hospital made this neither pragmatic nor possible.

We present the time from test to delivery based on the PlGF category (Figure 3) but did not include this in our primary outcome because timing of delivery is often resource or practitioner-dependent and not a reliable proxy for disease severity in the study setting.

This first study to evaluate whole blood POC-PlGF showed that it is feasible and accurate in a hospital in Sierra Leone, a country with high reported maternal (443/100 000^13^) and neonatal (3/1000^14^) mortality rates, challenging infrastructure (38% of health facilities lack reliable electricity^15^), and was acceptable (only 4 women refused). As diagnostic criteria for preeclampsia require resources often unavailable (eg, proteinuria dipsticks and ultrasound scans), we could not stratify analysis by suspected and confirmed preeclampsia although the mean BP was extremely high (173/118 mm Hg). PlGF was blinded to clinical teams, so care decisions, including delivery, were not influenced.

PlGF test performance has been demonstrated prospectively in LMIC settings; a Mozambique study found that abnormal PlGF in suspected preeclampsia was associated with higher risk of preeclampsia diagnosis, shorter time to delivery, and perinatal death.^8^ In a follow-up study, one-third of women with very abnormal PlGF had a stillbirth; the area under the receiver operating characteristic curve is 0.78 (95% CI, 0.70–0.86), with a sensitivity of 76.9% (95% CI, 60.7%–88.9%) and a specificity of 74.1% (95% CI, 68.0%–79.5%).^9^ In our study, with twice the number of stillbirths, both RONIA and Lepzi Quanti PlGF had higher sensitivity (98.8% and 95.8%) but more modest specificity (19.8% and 31.5%). A small prospective Indian study (n=50) found that abnormal SFlt-1/PlGF was significantly associated with severe preeclampsia (90.91% versus 8.00%; *P*<0.0001) and maternal complications (18.18% versus 0%; *P*=0.04) compared with 40.0% versus 8.0% in women tested <34 weeks of gestation in our study.^10^

All studies, to date, have used benchtop PlGF analyzers, requiring centrifugation, challenging in Sierra Leone and similar settings. PlGF alone and SFlt-1/PlGF ratio are both National Institute for Health and Care Excellence–recommended, with no differences in predictive ability^16,17^ However, POC testing with 2 assays is more complex and currently unavailable.

## Conclusions

This study indicates that both whole blood RONIA and Lepzi Quanti POC-PlGF testing could enable targeted management, avoiding unnecessary intervention with a normal result, and is feasible in challenging settings.

## Perspectives

This is the first prospective observational study of novel whole blood POC-PlGF testing. Women with a very abnormal PlGF had up to a 20% chance of death or eclampsia (PPVs of 14.4 and 20.3%) and would be routinely delivered based on these risks in well-resourced settings. However, if it is unlikely to happen, there is considerable benefit in avoiding preterm delivery in resource-limited settings, and therefore, high sensitivity and NPV, representing a reliable rule-out test, have important clinical utility. For women falling into the intermediate abnormal PlGF category, increased surveillance is justified to monitor progress. We think that the current results add considerable value to management as a significant minority (up to 26% of women with normal PlGF) would change management. This should be evaluated in prospective trials and is the subject of the current PAPAGAIO (Preterm preeclampsia: Placental Growth factor for reduction of Adverse Outcomes) trial in Brazil, India, and Sierra Leone using these POC tests. POC testing is likely considerably cheaper than current PlGF-based tests. Given the cost of existing devices and tests (>$2200/device; >$30 USD/test),^8^ it is likely that POC alternatives could be adopted globally.

## Article Information

### Acknowledgments

The authors thank the participants and members of the National Institute for Health and Care Research Capacity Research Innovation Building Maternity Systems Consortium. The RONIA™ platform, the reader and PlGF test used in this study was intended for research use only. Our feasibility study, performed with RONIA™ platform, provided information that will allow further development of the assay and the reader software measurement protocol.

### Author Contributions

All authors contributed to the conception and planning of the work. K. Kuhrt, R. Cole, and M. M’Bayoh contributed to carrying out the work. K. Kuhrt led the writing. All authors contributed to analysis and interpretation of the work, writing the article, final approval of this version, and agree to be accountable for all aspects of the work.

### Sources of Funding

This work was supported by the UK Medical Research Council (grant MR/T038594/1) and the National Institute for Health and Care Research (grant NIHR133232).

### Disclosures

None.

### Supplemental Material

Tables S1–S13

Figures S1 and S2

## Appendix

Pineapple Study Group

Professor Adetunji Adeniji, Matron Christiana Squire, Dr Rossetta Cole, Dr Moses M’Bayoh, Dr Victoria Wisman, Ms Zainab Bangura, Sister Edith Macauley, Mr Eric Macarthy-N’dolenge, Dr Sovula Haja, Dr Emmanuel Chinonso, Ms Suzanne Thomas, Dr Thomasia Weekes, Dr Davida Sesay, Dr Mariama Koker, and Ms Rebecca M’Bayoh.
